# Differences in miRNA Expression in Early Stage Lung Adenocarcinomas that Did and Did Not Relapse

**DOI:** 10.1371/journal.pone.0101802

**Published:** 2014-07-16

**Authors:** Mick D. Edmonds, Christine M. Eischen

**Affiliations:** Department of Pathology, Microbiology and Immunology, Vanderbilt University Medical Center, Nashville, Tennessee, United States of America; Deutsches Krebsforschungszentrum, Germany

## Abstract

Relapse of adenocarcinoma, the most common non-small cell lung cancer (NSCLC), is a major clinical challenge to improving survival. To gain insight into the early molecular events that contribute to lung adenocarcinoma relapse, and taking into consideration potential cell type specificity, we used stringent criteria for sample selection. We measured miRNA expression only from flash frozen stage I lung adenocarcinomas, excluding other NSCLC subtypes. We compared miRNA expression in lung adenocarcinomas that relapsed within two years to those that did not relapse within three years after surgical resection prior to adjuvant therapy. The most significant differences in mRNA expression for recurrent tumors compared to non-recurrent tumors were decreases in miR-106b*, -187, -205, -449b, -774* and increases in miR-151-3p, let-7b, miR-215, -520b, and -512-3p. A unique comparison between adjacent normal lung tissue from relapse and non-relapse groups revealed dramatically different miRNA expression, suggesting dysregulation of miRNA in the environment around the tumor. To assess patient-to-patient variability, miRNA levels in the tumors were normalized to levels in matched adjacent normal lung tissue. This analysis revealed a different set of significantly altered miRNA in tumors that recurred compared to tumors that did not. Together our analyses elucidated miRNA not previously linked to lung adenocarcinoma that likely have important roles in its development and progression. Our results also highlight the differences in miRNA expression in normal lung tissue in adenocarcinomas that do and do not recur. Most notably, our data identified those miRNA that distinguish early stage tumors likely to relapse prior to treatment and miRNA that could be further studied for use as biomarkers for prognosis, patient monitoring, and/or treatment decisions.

## Introduction

Lung cancer is the leading cause of cancer deaths worldwide. Non-small cell lung cancer (NSCLC) comprises 85% of lung cancers with adenocarcinoma its most common subtype [1]. Although early detection of NSCLC provides the greatest likelihood for five-year survival, the risk of relapse remains high after surgical resection and treatment [1]. Identifying the genes either responsible for or that could be used to predict tumors likely to relapse is essential, if improvements in disease survival are to be achieved.

MicroRNA (miRNA) are small, non-coding, RNA molecules that regulate gene expression typically by binding the 3′untranslated region (UTR) of mRNA [2]. This binding results in decreased expression of the mRNA. An individual miRNA can bind multiple distinct mRNA resulting in changes in complex cellular processes, such as signaling, growth, differentiation, and transformation [2]. Given that miRNA have such crucial cellular roles, their dysregulation have been reported in numerous cancer types, including NSCLC [2]. In cancer, specific miRNA are reported to function as powerful tumor suppressors or oncogenes [2]. They may also serve as important biomarkers for disease development and/or progression [2].

Several studies have reported links between miRNA expression and lung cancer outcomes; however, there is little overlap in the miRNA identified between these studies [3]−[8]. This lack of consensus may be attributed to technical reasons, such as: 1) the platform used (e.g., hybridization-, real-time PCR, or RNA-sequencing), 2) specimen type (e.g., flash frozen or FFPE), and/or 3) the method(s) for analysis of data sets. In addition, differences could also be due to biological reasons, such as the common approach of mixing histological subtypes (adenocarcinoma, squamous cell carcinoma, and large cell carcinoma,), analyzing tumors that have received different adjuvant therapies, and the grouping of early and late stage disease. Each of the aforementioned parameters can influence the levels of miRNA detected and thus, alter the miRNA determined to be significantly changed in adenocarcinoma development and disease recurrence. One of the most difficult variables to control for, that being patient-to-patient differences in miRNA expression, is also likely to contribute to differences between studies.

To address these challenges, we examined miRNA expression in NSCLC patients that did or did not recur from flash frozen, matched early stage lung adenocarcinoma using quantitative real-time PCR analysis. We identified specific changes in miRNA expression that distinguish tumors that recurred and did not non-recur from each other. Significant differences between the adjacent normal lung tissue from recurrent and non-recurrent patients were also identified. Normalization of patient-to-patient differences in miRNA expression with regression analysis identified miRNA that distinguished tumors that relapsed from those that did not. Our results indicate miRNA dysregulation occurs early in lung adenocarcinoma development and specific miRNA may be used to distinguish patients at risk for relapse prior to adjuvant treatments.

## Materials and Methods

### Patient samples

De-identified frozen human samples (lung adenocarcinoma and adjacent normal lung) collected from 2002−2009 were obtained from the Vanderbilt University Medical Center Lung Biorepository that banks samples following patient consent. We followed stringent criteria for sample selection for two cohorts. All samples were surgically resected prior to chemotherapy and/or radiotherapy and snap frozen. Tumors were all stage I (A or B) non-small cell lung adenocarcinoma. Samples came from patients that presented with relapsed lung adenocarcinoma within 2 years of resection, or were obtained from patients that had no evidence of recurrence 3 years post resection. All tumor samples were >80% tumor. Adjacent normal lung tissue for each patient was assessed where possible. Adjacent normal tissue was collected 2−4 cm from the adenocarcinomas. Hematoxylin and eosin stained sections for each normal sample were evaluated by a Board Certified Pathologist and determined to lack any pre-cancerous lesions. In the first cohort, adjacent normal lung tissue was available for 6 of 10 patients that did not relapse and 8 of 10 patients that relapsed. In the second cohort, adjacent normal lung tissue was available for 4 of 5 patients that did not relapse and 3 of 3 patients that relapsed. Specific patient demographics are indicated in Table 1.

**Table 1:** **Clinical characteristics of patients with lung adenocarcinoma in this study.**

Cohort	Patient	Gender	Age	Smoking History	Pack Years	Stage	Tumor Size (cm)	Year*	Relapsed in 2 yrs	Adjacent Normal
1	1	Male	73	Smoker	75	1B	1.8	2008	Yes	Yes
1	2	Male	55	Smoker	25	IA	2.1	2008	Yes	No
1	3	Male	67	Ex-smoker	100	IA	2.8	2008	Yes	Yes
1	4	Male	55	Ex-smoker	30	IB	5.4	2002	Yes	No
1	5	Female	69	Ex-smoker	35	IB	1.2	2007	Yes	Yes
1	6	Male	56	Ex-smoker	38	IB	6.2	2007	Yes	Yes
1	7	Male	64	Ex-smoker	30	IA	3.0	2004	Yes	Yes
1	8	Female	47	Smoker	30	1A	2.5	2004	Yes	Yes
1	9	Male	54	Ex-smoker	30	IB	7.1	2005	Yes	Yes
1	10	Male	74	Ex-smoker	100	IB	2.9	2005	Yes	Yes
1	11	Male	81	Ex-smoker	70	IA	1.2	2002	No	Yes
1	12	Male	78	Ex-smoker	62	IB	4.0	2002	No	No
1	13	Female	73	Ex-smoker	75	IB	1.2	2006	No	Yes
1	14	Female	72	Smoker	34	IB	0.7	2004	No	Yes
1	15	Male	90	Smoker	62	IB	4.0	2002	No	Yes
1	16	Female	51	Ex-smoker	33	IA	2.6	2008	No	Yes
1	17	Male	61	Ex-smoker	90	IB	3.9	2004	No	No
1	18	Female	65	Ex-smoker	40	IB	4.7	2002	No	Yes
1	19	Female	68	Ex-smoker	100	IB	1.1	2008	No	No
1	20	Male	54	Smoker	30	IB	3.0	2005	No	No
2	1	Female	68	Never Smoke	0	IB	3.0	2005	Yes	Yes
2	2	Male	57	Ex-smoker	40	IB	5.2	2009	Yes	Yes
2	3	Male	64	Ex-smoker	30	IA	3.0	2004	Yes	Yes
2	4	Female	73	Ex-smoker	15	IA	1.7	2008	No	Yes
2	5	Female	63	Ex-smoker	0.3	IA	2.6	2008	No	Yes
2	6	Male	61	Ex-smoker	80	IB	5.1	2008	No	No
2	7	Female	71	Ex-smoker	58	IB	1.1	2008	No	Yes
2	8	Male	69	Ex-smoker	40	IA	1.0	2008	No	Yes

*The year the tumor was resected from which the sample for analysis was obtained and frozen.

### Quantitative PCR (qPCR) array and quantitative real-time PCR (qRT-PCR)

Total RNA was extracted from frozen patient samples using Trizol as per manufacture' protocol. qPCR array analysis was conducted by Life Technologies. Briefly, cDNA was made using TaqMan MicroRNA Reverse Transcription Kit with Megaplex Primer Pools, Human Pools Set v3.0. miRNA cDNA were amplified using 2X TaqMan OpenArray real-time PCR Master mix and an OpenArray NT cycler System. All sequences were derived from miRBase v.14. To verify the array data, qRT-PCR on individual miRNA was performed on the same samples and samples from a second patient cohort (see above). Specifically, cDNA was synthesized using TaqMan MicroRNA Reverse Transcription Kit and TaqMan miRNA specific miRNA RT primers. cDNA levels were measured in triplicate using TaqMan Universal PCR Master Mix, miRNA TaqMan primer probes, and an ABI PRISM 7000 Sequence Detection System. All data were analyzed using ABI PRISM 7000 SDS software v1.2.3. All reagents and equipment were from Life Technologies (Carlsbad, CA).

### Data and Statistical Analysis

For qPCR arrays, OpenArray NT cycler ncx files were imported into Data Assist v.3.0, and Ct values generated. miRNA expression was normalized to endogenous control using the following formula: 2^−[Ct(miRNA)-median(CtRNU48)]^. RNU6B, RNU24, RNU44, RNU46, RNU48, and miR-126 expression were evaluated for potential use as endogenous small RNA controls. RNU48 was selected as the endogenous small RNA control for all comparisons after determining its expression demonstrated the least sample-to-sample variability. qPCR data were log2 transformed to normally distribute data for the software package limma [9], which has been previously reported to accurately identify differentially expressed miRNA in qPCR studies [10], [11]. Limma was used to calculate fold change and p values. For comparison of two groups, fold change for each normalized miRNA was calculated as: log_2_ (mean value for group A/mean value for group B). For TaqMan qRT-PCR experiments, miRNA expression was normalized as follows: 2^−[meanCt(miRNA)-meanCt(RNU48)]^, and student's t-tests were used to determine significance between comparison groups.

### Ethic Statement

The Vanderbilt Institutional Review Board approved the banking of patient samples. Written consent was obtained from the patients.

## Results and Discussion

A comprehensive evaluation of miRNA in lung adenocarcinoma and adjacent normal lung tissue from patients was conducted using TaqMan qPCR miRNA arrays. miRNA probes used were specific for mature miRNA. This approach avoids detection of miRNA precursor forms (pri- and pre-), which can have altered expression in human malignancies that do not necessarily correlate to mature miRNA levels and can confound results [12]. Furthermore, our method also avoids issues with the detection of lower copy number miRNA, the variability associated with different melting temperatures, and the differences in hybridization efficiencies between probe/miRNA pairs that exist with miRNA hybridization arrays. Levels of 754 miRNA were obtained from frozen, resected stage I lung adenocarcinoma from patients that relapsed within 2 years of resection, patients that did not relapse within 3 years of resection, and adjacent normal lung tissue from both. Due to the stringent criteria used to select samples, this limited the number of samples available to evaluate. miRNA expression data were evaluated and normalized to an endogenous small RNA control, and then six different comparisons between the groups were performed.

### miRNA differences between adjacent normal and stage I lung adenocarcinoma tumors

**Figure 1:**
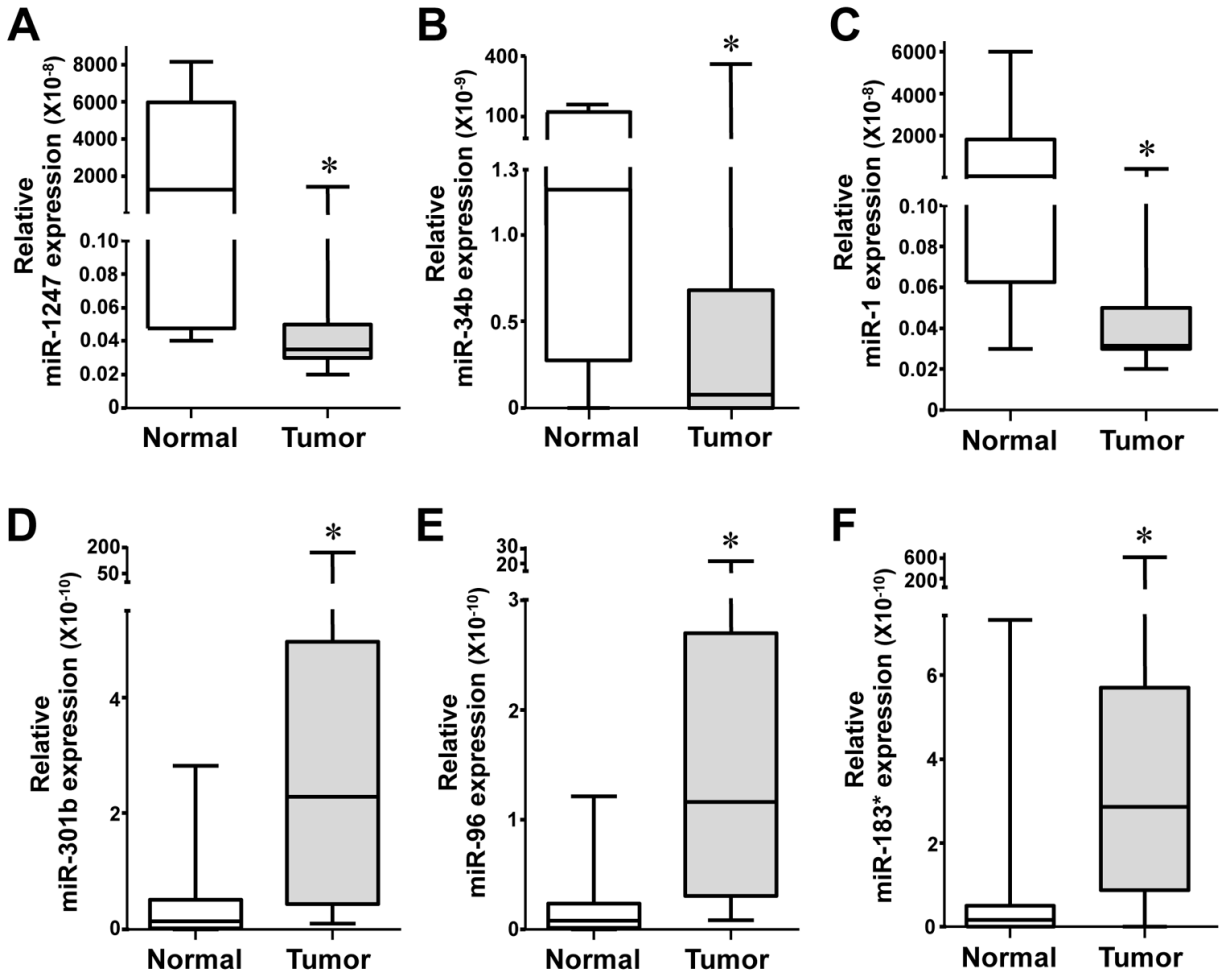
Overexpression or reduced expression of specific miRNA in stage I lung adenocarcinoma. miRNA expression for the indicated miRNA was determined by TaqMan qPCR array in individual patient samples of lung adenocarcinoma (n = 20) and adjacent normal lung tissue (n = 14). ΔCt values graphed are relative to RNU48, an endogenous small RNA control. The box represents the first and third quartiles and the horizontal line within the box is the median. Whiskers represent the maximum and minimum values. *p values listed in Table 2A.

The first comparison evaluated miRNA expression differences between all adjacent normal lung tissue samples and all lung adenocarcinoma tumor samples, which included both tumors that did and did not relapse. In the tumors, the most significantly decreased miRNA were miR-133b, -1247, -34b, -139-3p, and -1, and the most significantly increased miRNA were miR-301b, -96, -1269, -147b, and -183* (Table 2A and Fig 1A–F). Of these miRNA, miR-1247 (Fig 1A), -301b (Fig 1D), -1269, and -147b have not been previously reported in lung cancer, and their functions remain unknown. Our results on the well known miRNA, miR-34b (Fig 1B), are analogous to those previously reported [13]. A lesser known miRNA, miR-133b, also appears to contribute to lung cancer. Ectopic miR-133b expression induces apoptosis, inhibits cell growth, migration, invasion, and enhances sensitivity to gefitinib in NSCLC adenocarcinoma cell lines [14], which may explain why we detected decreased levels in lung adenocarcinomas in our analyses. Similarly, reduced miR-1 expression we detected (Fig 1C) was also previously observed and shown to have anti-oncogenic properties in NSCLC [15]. In contrast to our results, increased miR-139-3p was reported in lung squamous cell carcinoma (SCC), bladder carcinoma, and plasma of patients with metastatic colorectal carcinoma (CRC) [16], [17].

**Table 2:** **Comparisons of miRNA expression between lung adenocarcinomas that did or did not recur and adjacent normal lung tissue.**

A. All Tumor (n = 20) compared to all Normal (n = 14):					
miRNA	Fold Change	P	miRNA	Fold Change	P
hsa-miR-133b	−9.2	1.24E-04	hsa-miR-301b	5.3	9.1E-04
hsa-miR-1247	−8.9	4.46E-04	hsa-miR-96	3.8	0.001
hsa-miR-34b	−8.3	0.001	hsa-miR-1269	8.3	0.001
hsa-miR-139-3p	−8.3	0.003	hsa-miR-147b	6.4	0.004
hsa-miR-1	−7.7	0.006	has-miR-183*	7.3	0.005

B. Recurrent Tumor (n = 10) compared to Non-Recurrent Tumor (n = 10):					
miRNA	Fold Change	P	miRNA	Fold Change	P
hsa-miR-106b*	−9.3	0.002	hsa-miR-151-3p	8.1	0.008
hsa-miR-187	−9.1	0.005	hsa-let-7b	11.0	0.009
hsa-miR-205	−7.3	0.007	hsa-miR-215	11.9	0.009
hsa-miR-449b	−6.9	0.010	hsa-miR-520b	7.5	0.010
hsa-miR-744*	−6.2	0.025	hsa-miR-512-3p	7.7	0.010

C. Non-Recurrent Tumor (n = 6) compared to matched Non-Recurrent Adjacent Normal (n = 6):					
miRNA	Fold Change	P	miRNA	Fold Change	P
hsa-miR-139-5p	−2.9	0.001	hsa-miR-193b*	9.4	0.010
hsa-miR-451	−4.7	0.003	hsa-miR-9*	5.5	0.010
hsa-miR-133a	−2.9	0.005	hsa-miR-708	1.9	0.010
hsa-miR-126	−2.4	0.012	hsa-miR-301b	7.0	0.012
hsa-miR-338-3p	−3.3	0.013	hsa-miR-193b	1.4	0.013

D. Recurrent Tumor (n = 8) compared to matched Recurrent Adjacent Normal (n = 8):					
miRNA	Fold Change	P	miRNA	Fold Change	P
hsa-miR-1247	−16.6	4.34E-10	hsa-miR-147b	10.1	0.002
hsa-miR-34b	−10.1	1.51E-04	hsa-miR-550	2.3	0.020
hsa-miR-432	−7.2	0.001	hsa-miR-589	2.9	0.020
hsa-miR-551b	−8.5	0.001	hsa-miR-372	6.3	0.027
hsa-miR-30d*	−10.1	0.001	hsa-miR-1269	6.0	0.046

E. Recurrent Adjacent Normal (n = 8) compared to Non-Recurrent Adjacent Normal (n = 6):					
miRNA	Fold Change	P	miRNA	Fold Change	P
hsa-miR-501-3p	−5.1	0.037	hsa-miR-454*	4.3	0.001
hsa-miR-106b*	−4.2	0.018	hsa-let-7i*	7.6	0.001
hsa-miR-1256	−3.3	0.012	hsa-miR-26a-1*	7.8	0.001
hsa-miR-184	−2.7	0.006	hsa-miR-432	8.9	0.001
hsa-miR-618	−1.7	0.047	has-miR-331-5p	9.1	0.001

miRNA expression was normalized to endogenous control RNU48 and fold change and p values of the differences were calculated by limma [log2(mean value group A/mean value group B)] [9].

miR-96 and -183* are members of the miR-183 polycistronic family that consist of miR-96, and -182, and -183. Meta-analysis of miR-183 family expression in human cancer studies revealed increased expression of at least one member of this miRNA family in lung cancer as well as bladder cancer and hepatocellular carcinoma (HCC) [18]. One study, which examined three late stage NSCLC adenocarcinoma samples, found that miR-96 was significantly up-regulated [19]. Our data showed miR-96 was significantly overexpressed already in stage I lung adenocarcinoma (Fig 1E). Additionally, we detected significantly increased miR-183* levels in lung adenocarcinoma (Fig 1F).

### Differences in miRNA expression in non-recurrent lung adenocarcinoma compared to recurrent lung adenocarcinoma

We also compared miRNA expression differences between tumors that recurred within 2 years or did not recur in 3 years after resection. In this comparison, the most significantly decreased miRNA in the recurrent tumors were miR-106b*, -187, -205, -449b, and -774* and the most significantly increased miRNA were miR-151-3p, let-7b, miR-215, -520b, and -512-3p (Table 2B). Currently, there are no reports as to the role of miR-774*, -151-3p, and -512-3p in lung cancer. Surprisingly, miR-106b*, which is a paralog to the oncogenic polycistronic miR-17-92 family that is overexpressed in numerous human cancers, including lung [20], was decreased. Equally unexpected were increased levels of let-7b and miR-215 in the recurrent, presumably more aggressive, tumors. Reduced let-7b correlates to worse NSCLC survival outcomes [21], and miR-215 is a p53-induced miRNA that suppresses colon cancer cell growth [22]. In recurrent tumors, we also detected decreased levels of miR-187 and miR-205, which were shown to be oncogenic in breast cancer and NSCLC, respectively, *in vitro* [23], [24]. miR-520b is a family member to miR-520c, which is increased in human breast carcinoma and promotes invasion and metastasis of breast cancer cells [25]. A bioinformatic approach reported that a subset of increased miR-449b targets, presumably due to decreased miR-449b, along with miR-34b/c levels, served as a prognostic marker for lung adenocarcinoma [26].

### miRNA expression differs between non-recurrent adenocarcinoma and matched adjacent normal tissue

For our third comparison we examined miRNA expression differences between matched adjacent normal tissue and lung adenocarcinoma tumors that did not recur within 3 years. The most significantly decreased miRNA in tumors that did not recur were miR-139-5p, -451, -133a, -126, and -338-3p, and the most significantly increased miRNA were miR-193b*, -9*, -708, -301b, and -193b (Table 2C). Of the ten most significantly altered miRNA in tumors that did not recur, all but miR-301b have been previously linked to lung cancer. Specifically, miR-139-5p, -451, and -126 were decreased in human NSCLC [27]–[29], and miR-451, -133a, -126, and -193b have tumor suppressing properties in lung cells [5], [30], [31]. miR-139-5p was also reported decreased in the sputum of lung squamous cell carcinoma and serves as a biomarker for early detection [27]. miR-9* is a transcriptional target of oncogenic c-Myc [32], and a miRNA that we recently identified as overexpressed in early stage NSCLC [33]. Increased miR-708 expression is associated with poor survival in lung NSCLC adenocarcinoma [34], and we detect increased levels in adenocarcinomas that relapsed (Table 2C).

### miRNA expression is different in adenocarcinomas that relapse compared to matched adjacent normal tissue

The next comparison examined miRNA expression differences between matched adjacent normal tissue and lung adenocarcinoma tumors that recurred within 2 years. Two of the most significantly decreased miRNA (miR-1247 and -34b) and two of the most significantly increased miRNA (miR-147b and -1269) in recurrent tumors were also identified in tumors in our first comparison between all normal tissue and all tumors (Table 2A and 2D). The other most significantly decreased miRNA in recurrent tumors were miR-432, -551b, and -30d*, and the most significantly increased miRNA were miR-550, -589, and -372 (Table 2D). miR-432, -551b, -30d,* and -589 have not been previously implicated in lung cancer. Increased miR-550 levels have been reported in NSCLC adenocarcinoma patients [35]. In addition, miR-372 is oncogenic in human testicular germ cell tumors, and increased expression associates with poor prognoses in glioma, HCC, and CRC. Increased sputum miR-372 levels, together with other miRNA (miR-21, miR-143, miR-155, miR-210), were biomarkers used to detect early NSCLC adenocarcinoma [36].

### miRNA differs in adjacent normal lung tissue in patients that did and did not recur

**Figure 2:**
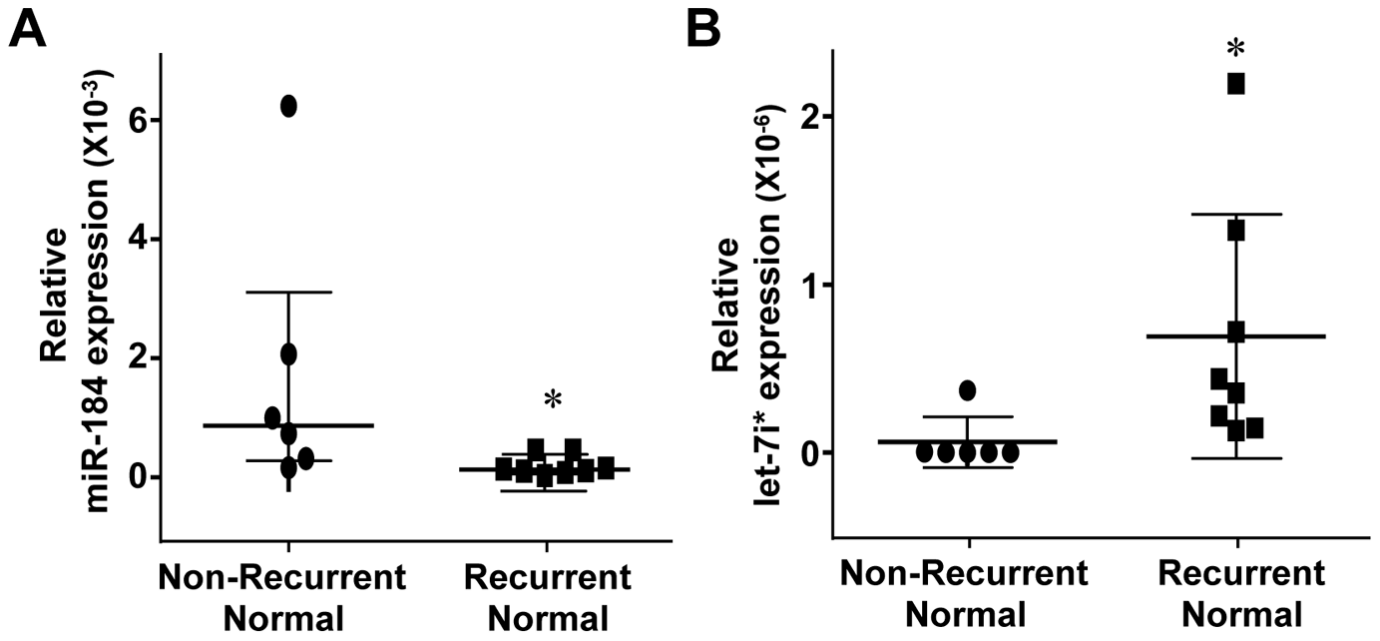
miRNA differences in normal adjacent lung tissue from lung adenocarcinoma patients that went on to relapse and those that did not. miRNA expression for miR-184 and let-7i* was measured by TaqMan qPCR array and relative ΔCt values are plotted. Each symbol represents an individual patient sample. Median of both groups graphed with interquartile range. *p values listed in Table 2E.

We performed one comparison, which is rarely done, between adjacent normal lung tissue from the adenocarcinoma patients that recurred and that did not recur, and it revealed striking differences between the two. We included this comparison given emerging evidence indicating a significant role of surrounding adjacent stroma, and more recently to adjacent normal tissue, in the development and progression of tumors [37]–[39]. The most significantly decreased miRNA in the normal lung tissue from patients that relapsed were miR-501-3p, -106b*, -1256, -184, and -618, and the most significantly increased miRNA were miR-454, -7i*, -26a-1*, -432, and -331-5p (Table 2E and Fig 2). Notably, miR-106b*, a paralog to the oncogenic miR-17-92 cistron, was decreased in the normal lung tissue from patients whose lung adenocarcinoma relapsed and in the tumors from these same patients compared to their respective counterparts in patients that did not relapse (Table 2B and 2E). Additionally, miR-432 levels were increased in normal lung tissue from patients that relapsed compared to the tumors in those patients (Table 2D) and also when compared to normal lung tissue from patients that did not recur (Table 2E). miR-1256 has not been linked to lung cancer, but its up-regulation leads to increased growth, migration, and invasion of prostate cancer cells [40]. We detected reduced levels of miR-184 (Fig 2A), but it is reported to have oncogenic function in HCC and is overexpressed in human glioma and SCC of the tongue [41]. We detected increased expression of let-7i* (Fig 2B), a member of the let-7 family, which are negative regulators of Ras, a key oncogenic driver of NSCLC adenocarcinomas, and let-7i has been previously reported to classify human NSCLC subtypes from transthoracic needle specimens [42]. Increased miR-618 and decreased miR-454* expression have been reported in esophageal cancer [43], [44]. Given the rarity of this comparison, it is not surprising that many of the most significant miRNA changes we detected in comparison of normal lung tissue have not been reported in lung cancer, and these include miR-501-3p, -618, -454*, -26a-1*, and -331-5p.

### Analysis of miRNA differences in a second patient cohort

**Figure 3:**
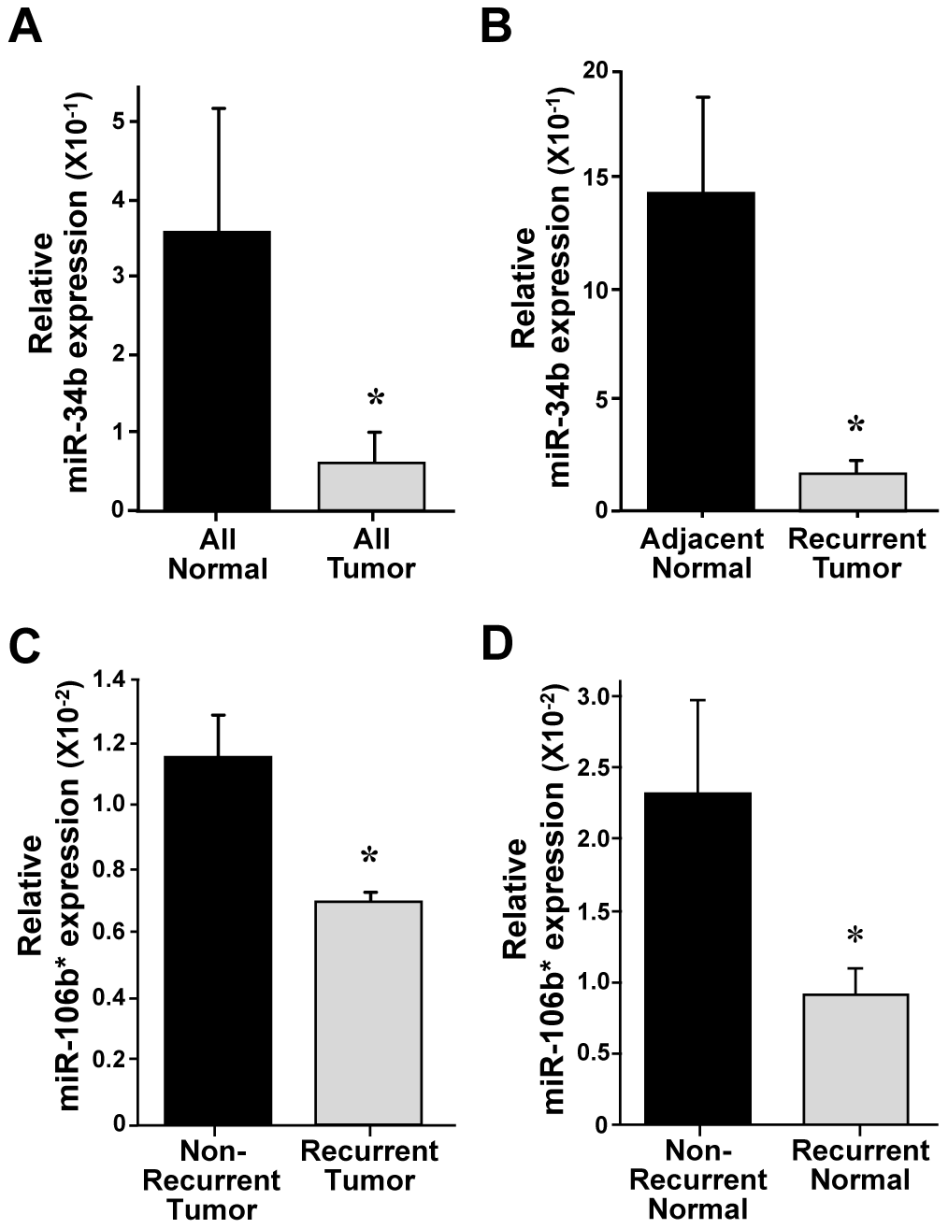
Validation of reduced miR-34b and miR-106b* levels in stage I lung adenocarcinoma and normal adjacent lung tissue. miR-34b and miR-106b* expression was measured using TaqMan qRT-PCR in a second cohort of stage I lung adenocarcinoma and adjacent normal lung tissue. ΔCt values graphed are relative to the endogenous small RNA control RNU48 with SEM. (A) Mean relative ΔCt value of tumors that did and did not relapse (n = 8) compared to mean value of adjacent normal lung tissues for both tumors (n = 7), *p = 0.03. (B) Mean relative ΔCt values of recurrent lung adenocarcinoma (n = 3) or adjacent normal lung tissue (n = 3), *p = 0.02. (C) Mean relative ΔCt values in tumors that did (n = 3) or did not (n = 5) relapse, *p = 0.02. (D) Mean relative ΔCt values of adjacent normal lung tissue from patients that did (n = 3) or did not relapse (n = 4), *p = 0.05. All p values calculated by student's t-test.

Although samples for the first patient cohort were subjected to qRT-PCR for select miRNA to verify the array data, a second smaller patient cohort that met the same criteria as our first cohort was specifically obtained and evaluated. We assessed expression of miRNA that had significantly altered expression in multiple comparisons from the qPCR arrays. Our initial cohort had identified miR-34b as being decreased in lung adenocarcinoma tumors compared to levels in normal lung (Table 2A and Fig 1B). Using qRT-PCR, a significant decrease was also detected in lung adenocarcinomas in our second cohort (Fig 3A). We also identified in our first cohort that miR-34b had a significant decrease in the lung tumors that came from patients that went on to relapse when compared to their respective adjacent normal lung tissue (Table 2D), and the same was detected in our second cohort (Fig 3B). When comparing miRNA between tumors that did and did not recur in our first patient cohort, we determined miR-106b* was significantly decreased in tumors that relapsed (Table 2B). We obtained an analogous result in our second cohort, with a decrease in miR-106b* in the tumors that recurred (Fig 3C). Data from the first cohort also revealed miR-106b* was decreased when comparing adjacent normal tissue from patients that did relapse to the adjacent normal tissue from those that did not relapse (Table 2E). Similar results were detected in our validation cohort (Fig 3D). These results suggest the surrounding tumor stroma is altered along with the tumor itself.

### Normalizing miRNA expression in tumors to adjacent normal tissue reveals differences in miRNA expression

**Figure 4:**
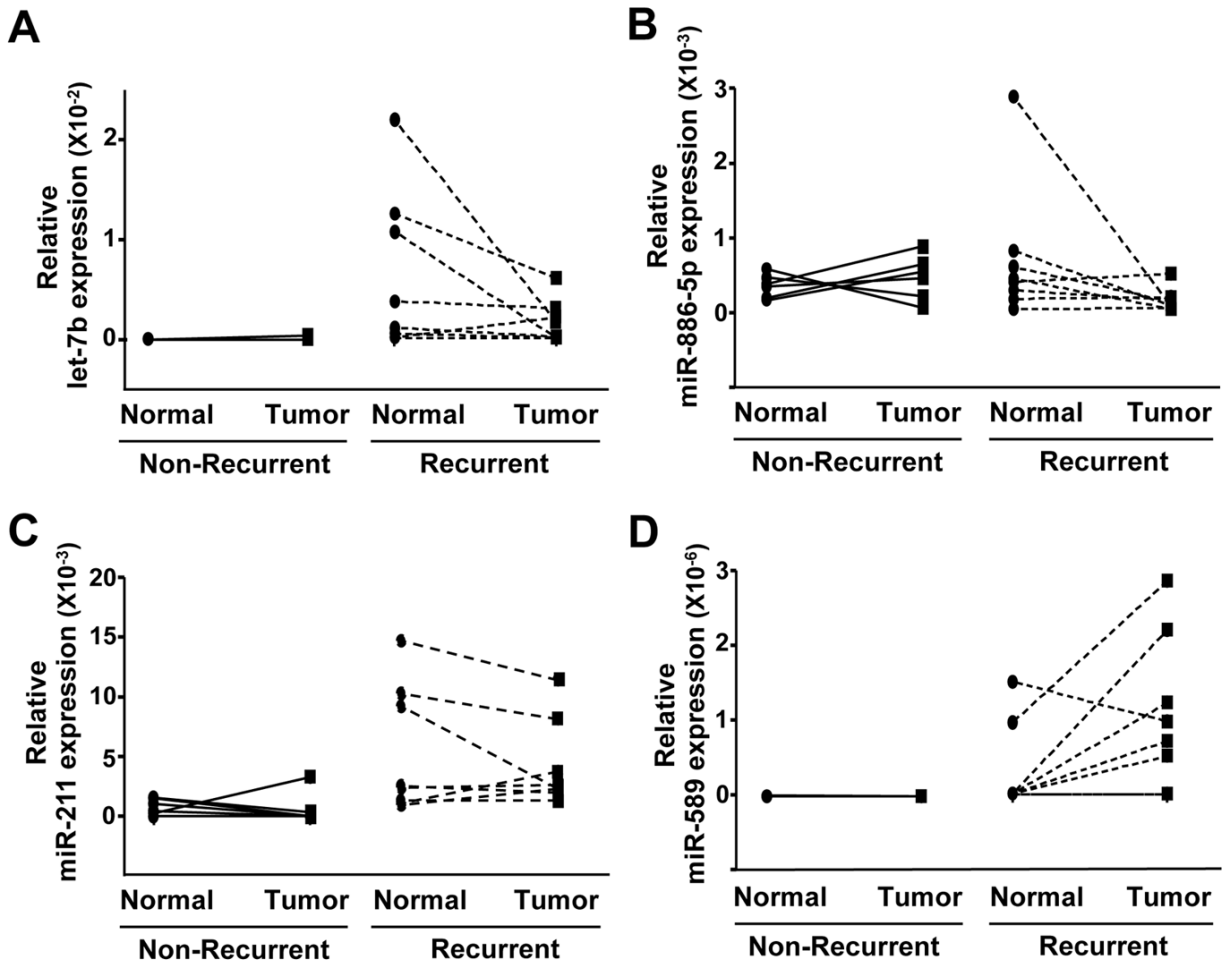
Reduced let-7b and -886-5p and increased miR-211 and -589 are potential biomarkers for stage I lung adenocarcinomas that recur. miRNA expression for let-7b, miR-886-5p, -211, and -589 was determined by TaqMan qPCR arrays in lung adenocarcinoma tumors that relapsed (n = 8) or that did not relapse (n = 6) and normal adjacent lung tissue for both (n = 8 and 6, respectively). ΔCt values graphed are relative to small RNA endogenous control RNU48. Lines connect patient matched samples.

Based on the data described above and particularly the differences in miRNA observed in normal tissue between patients that relapsed and those that did not, we used regression analysis to evaluate our data. Regression analysis is a method whereby the qPCR array expression for each miRNA in an adenocarcinoma tumor is normalized to the expression in its corresponding matched adjacent normal lung tissue. Data evaluated in this manner controls for patient-to-patient variance in miRNA expression, and this analysis led to the largest increases and decreases detected in miRNA expression in this study. The most significant decreases in miRNA in lung tumors that eventually recurred compared to those that did not were let-7b, miR-106b*, -187, -650, and -886-5p, and the most significant increases were in miR125a-3p, -589, -450a, -211, and -139-3p (Table 3 and Fig 4). As stated previously, the let-7 family, of which let-7b is a member, negatively regulates Ras, and let-7b expression correlates to adenocarcinoma progression free survival, but not overall survival [21]. Low expression of let-7b before adjuvant therapy has been shown to correlate with poor response to chemotherapy in esophageal squamous cell carcinoma [45]. Our comparison in Table 2B revealed a 10 fold up-regulation of let-7b in the tumors that relapsed compared to those that did not, as tumors that did not relapse had very low levels of let-7b. (Table 2B and Fig 4A). However, let-7b levels in adjacent normal lung tissue samples were almost undetectable in the non-relapse adjacent normal tissue and higher in the relapse adjacent normal tissue (Fig 4A), explaining why when the normal tissue levels were used to normalize let-7b, the tumors that relapsed showed reduced levels (Table 3). Again, we detected decreased levels of miR-106b* in this analysis. Interestingly, miR-139-3p showed decreased expression in tumors overall (those that did and did not recur combined) when compared to normal lung tissue (Table 2A), but this analysis determined that tumors that relapse express significantly more miR-139-3p than those that do not relapse (Table 3). Decreased miR-187 was reported in clear cell renal and prostate carcinomas, yet it promotes breast carcinoma invasive potential [23], [46], [47]. miR-886-5p, which was decreased in recurrent tumors in our analyses (Fig 4B), has recently been re-defined as a vault RNA, as it was shown to associate with the vault ribonucleoprotein complex, which have long been associated with multi-drug resistance in malignant cells [48]. miR-125a-3p induces apoptosis, prevents growth, and suppresses migration and invasion in lung cancer cells [49], [50], and miR-450a promotes epithelial to mesenchymal transition in human endometrial carcinoma [51]. miR-211 was increased in recurrent tumors (Fig 4C), and it is considered a pro-survival miRNA that promotes CRC cell growth and head and neck carcinoma progression [52], [53]. Increased miR-211 expression also correlates to poor prognosis in oral carcinoma [54]. It was reported that miR-650 was significantly higher in NSCLC adenocarcinoma tissues than in corresponding non-tumor tissues, and high expression of miR-650 significantly associated with lymph node metastasis and poor prognosis for NSCLC adenocarcinoma patients [55]. The sample set used in that study was a mix of stage 1-IV samples with over half of the samples being late stage. Our data show that stage I adenocarcinoma with decreased miR-650 relapsed within two years from resection.

**Table 3:** **Regression analysis of miRNA in non-recurrent and recurrent tumor relative to their normal adjacent tissue.**

Recurrent Tumor (n = 8) compared to Non-Recurrent Tumor (n = 6):					
miRNA	Fold Change	P	miRNA	Fold Change	P
hsa-let-7b	−48.7	0.027	hsa-miR-125a-3p	12.4	0.0001
hsa-miR-106b*	−9.7	0.017	hsa-miR-589	4.6	0.0015
hsa-miR-187	−7.8	0.036	hsa-miR-450a	6.2	0.0044
hsa-miR-650	−4.7	0.049	hsa-miR-211	13.8	0.0117
hsa-miR-886-5p	−1.7	0.048	hsa-miR-139-3p	10.9	0.0128

After normalizing miRNA expression in each adenocarcinoma to endogenous control RNU48, they were normalized to the expression in the corresponding matched adjacent normal control lung tissue. Limma [9] was then used to identify differentially expressed miRNA between tumors that did and did not recur and calculate p values.

## Conclusions

Dysregulation of miRNA has been demonstrated in nearly every cancer type, including lung adenocarcinoma [2]. Alterations in miRNA expression can be a consequence of or contribute to tumorigenesis and/or tumor progression [2]. miRNA function and expression in human cancer is complicated and its context specificity is just beginning to be elucidated [56]. Lung cancers classified under NSCLC, which its most common subtypes include adenocarcinoma and squamous cell carcinoma, are at the cellular and molecular level distinct [57]. Accordingly, when miRNA expression is examined, significant differences are detected between NSCLC subtypes [58]. Given the tissue and context specific nature of miRNA, we focused our studies only on stage I lung adenocarcinoma prior to treatment. Our analyses revealed new insight into lung adenocarcinoma, showing miRNA not previously linked to lung cancer and importantly, tumors that relapsed had significantly different miRNA expression than those tumors that do not relapse. We also determined that miRNA expression in normal lung tissue that is adjacent to tumors that recur are different than in normal lung tissue that is adjacent to tumors that do not recur. Our data indicate miRNA analysis in early stage lung adenocarcinoma may be a helpful biomarker for identifying those patients that have a likelihood of relapsing.

Our initial analyses examined miRNA with significantly altered expression in stage I lung adenocarcinoma regardless if it relapsed or not, and it revealed several novel changes. Some of these changes include decreases in miR-34b and -1247 and increases in family members of the miR-183 polycistronic family and miR-301b. To date, there are no reports for miR-1247 and -301b in human lung cancer, whereas miR-34b has recently been linked to early NSCLC adenocarcinoma development [59]. Increased expression of the miR-183 family (miR-96, -182, and -183) was implicated in NSCLC as a potential biomarker for both NSCLC adenocarcinoma and squamous cell carcinoma [19]; however, we detected two specific family members, miR-96 and -183*, significantly up regulated in stage I lung adenocarcinoma, and this has not been previously demonstrated. The 3′ strands of the miR-183 family have not been previously reported to have roles in NSCLC adenocarcinoma or in other cancers. Previous studies examining miRNA expression have pooled both lung squamous cell carcinoma and adenocarcinoma and have included tumors from early and late stage disease in their evaluation. While these approaches have certainly identified miRNA that are universally altered in lung tumors, they are unlikely to reveal alterations in miRNA that are context (tissue and stage) specific. Given our analyses were only on stage I adenocarcinoma, this may account for why we detected altered expression of specific miR-183 family members and not the whole family. Currently the transcriptional and post-transcriptional regulation of polycistronic miRNA is unclear; however, based on our data, as well as the data of others [19], members of the same cistron are not always equally expressed. Our data indicate that analysis of specific tissue and stage of disease can reveal those miRNA that are unique to that circumstance. Therefore, although miR-183 family overexpression is clearly involved in NSCLC, we demonstrate that the overexpression of two specific members may be important in early adenocarcinoma.

Our data also showed a significant decrease in miR-34b expression in stage I lung adenocarcinoma tumors. miR-34b, a member of the miR-34 family that also includes miR-34a and -34c, is regulated by the p53 tumor suppressor, induced upon oncogenic stress, and previously shown to be decreased in a cohort of mixed adenocarcinoma and squamous cell NSCLCs [59]. We detected higher levels of miR-34b in the tumors that did not recur compared to those that did, but levels in both were reduced when compared to normal lung tissue. The miR-34b promoter has been reported to be hyper-methylated in approximately half of lung adenocarcinomas [59]. Additionally, miR-34b is a transcriptional target of p53, which is inactivated in half of all NSCLC. Increased expression of miR-34b correlated to extended disease free survival of NSCLC patients [59], whereas its family member, miR-34a, is already a reported prognostic marker of relapse in NSCLC [60]. Our data suggest miR-34b levels are lowest in early stage tumors that relapse, and therefore, maybe able to be used to predict relapse of lung adenocarcinoma.

Unexpectedly, our initial analyses showed a decrease in miR-106b* in recurrent adenocarcinoma when compared to non-recurrent tumors. This decrease in miR-106b* was surprising as it is a member of the miR-106b-25 cluster and is a paralog to the miR-17-92 cluster, which is an oncogenic miRNA cluster [2]. Despite this, we also observed the decrease in miR-106b* in our second cohort, providing additional evidence that this occurs. In addition, as with miR-34b, we also determined miR-106b* is significantly decreased in the matched adjacent normal tissue from adenocarcinoma that relapsed. Future studies are needed to determine the reason for this decrease in miR-106b* in tumors that go on to relapse and its target mRNA that mediate its function.

One of the most notable changes we observed was the difference in miRNA expression between adjacent normal tissue from patients that went on to relapse and those that did not relapse. Notably, the miRNA significantly upregulated or downregulated between the two normal groups was substantially different from the miRNA identified in the comparison of the tumors from the two groups, except miR-106b*. Overall, the miRNA data on the normal lung tissue indicate there are specific changes unique to the adjacent normal tissue in patients that relapse from those that do not relapse. These differences provide important insight to the molecular changes in tumor stroma in lung tumors that do and do not relapse. Our data suggest the environment around the tumor likely contributes to the growth, survival, and/or progression of the tumor, as has been reported for multiple cancer types including lung [61], or is responding to the tumor and is just a bystander effect. Research will be needed to distinguish these and determine the contribution of altered miRNA in adjacent normal tissue to lung tumorigenesis.

The use of matched (adenocarcinoma and normal) patient sample sets offers powerful biologic insight that can be lost when samples are pooled and analyzed as mean values. Our normalization of the levels of each miRNA in the adenocarcinoma to its corresponding adjacent normal miRNA recapitulated our findings of some of the miRNA without matching the samples, such as the decrease in miR-106b* and miR-187. However, the analysis also revealed five new highly significant changes in miR-650, -886, -125*, -450, and -211 for which there are no previous reports on their association with lung adenocarcinoma or adenocarcinoma relapse, and four miRNA (miR-1247, -1269, -147b, and -301b) for which there are no reports in early lung adenocarcinoma. The normalization of the expression of each miRNA assayed in the adenocarcinoma to its corresponding adjacent normal tissue also produced other novel, unexpected results, one of which appeared to contradict an earlier analysis we performed. The analysis conducted comparing miRNA expression in adenocarcinoma from non-relapse and relapse tumor sets without normalizing to levels of each miRNA in adjacent normal tissue (Table 2B) showed an increase in let-7b, an antagonist to Ras signaling, in the tumors that relapsed [62]. We expected that such a miRNA would have been reduced in patients with more aggressive tumors; however, regression analysis determined that there was very low or undetectable expression of let-7b in both adjacent normal and adenocarcinoma tissue from the non-recurrent sample sets, whereas recurrent tissues had much higher basal levels of let-7b. Once the miRNA levels were normalized to their respective adjacent normal lung tissue, levels of let-7b were now significantly decreased in tumors that recurred. Overall, our studies provide new insights into the changes of miRNA expression in early adenocarcinoma development and adenocarcinoma relapse. Our results also provide novel miRNA associated with these processes that may be able to be used in the future for diagnosis, prognosis determination, patient monitoring, and/or treatment decisions.
